# Aortic prosthetic size predictor in aortic valve replacement

**DOI:** 10.1186/s13019-021-01601-z

**Published:** 2021-08-04

**Authors:** Anh Tuan Vo, Tomomi Nakajima, Trang Thi Thu Nguyen, Nguyen Thoi Hai Nguyen, Nga Bich Le, Tri Huu Cao, Dinh Hoang Nguyen

**Affiliations:** 1 Department of Cardiovascular Surgery, University Medical Center, University of Medicine and Pharmacy at Ho Chi Minh City, 215 Hong Bang Street, District 5, Ho Chi Minh City, Vietnam; 2 Department of Cardiovascular Surgery, Faculty of Medicine, University of Tsukuba, Tsukuba, Japan; 3 Department of Thoracic and Cardiovascular Surgery, Faculty of Medicine, University of Medicine and Pharmacy at Ho Chi Minh City, Ho Chi Minh City, Vietnam; 4 Department of Surgery, Nguyen Tri Phuong Hospital, Ho Chi Minh City, Vietnam

**Keywords:** Prosthetic size predictor, Patient-prothesis mismatch, Computed tomography, Transthoracic echocardiography

## Abstract

**Background:**

Patient-prosthesis mismatch (PPM) is a major concern in aortic valve replacement (AVR) and leads to perioperative morbidity and rehospitalization. Predicting aortic annulus diameter pre-procedurally is crucial to managing patients with high-risk of PPM.

**Objectives:**

To compare preoperative measurements of aortic annulus from echocardiography and CT scan with surgical sizing and develop an imaging-based algorithm to predict PPM.

**Methods:**

From January 2017 to December 2020, patients underwent AVR at a teaching hospital were examined. The relationship between imaging measurements with operative values was assesed using scatter plots and Pearson’s correlation coefficient. Univariable linear regression was then used to build the predictive model.

**Results:**

A total of 144 patients underwent AVR during the study period. Suture types and surgical approaches were not significantly associated with prosthesis size. CT scan-based measurements showed strong correlation with prosthesis size: mean diameter (R = 0.79), perimeter-derived diameter (R = 0.76), and area-derived diameter (R = 0.75). Mechanical valve and tissue valve shared similar correlation coefficients. Prosthesis size predictive models based on CT scan were 12.89 + 0.335 × d for mean diameter, 13.275 + 0.315 × d for perimeter-derived diameter and 13.626 + 0.309 × d for area-derived diameter.

**Conclusions:**

Preoperative CT scan measurements are a reliable predictor of aortic prosthesis size. Transthoracic echocardiography is a possible alternative, though it is highly performer-dependent and unable to represent the aortic annulus fully. Together, these two imaging modalities can be used to quantitatively anticipate PPM preoperatively.

## Introduction

Aortic valve replacement (AVR) remains the gold standard for patients with valvular lesions like aortic stenosis. A major complication following AVR is patient-prosthesis mismatch (PPM), a nonstructural dysfunction. First described in 1978 [1], PPM occurs when excessive pressure gradient is generated across a normally functioning prosthetic valve. Its severity is determined by the indexed effective orifice area (EOAi) as follows: not clinically significant (none or mild) when > 0.85 cm^2^/m^2^, moderate when between 0.65 and 0.85 cm^2^/m^2^, and severe when ≤ 0.65 cm^2^/m^2^.

While the negative impact of PPM in the early recovery period is controversial, it generally increases perioperative morbidity and rehospitalization due to heart failure and lack of left ventricular mass regression, and eventually long-term mortality [2, 3]. Several factors were found to increase the likelihood of PPM, including female sex, younger age, high body surface area (BSA), left ventricular end systolic diameter, aortic root dimension [4], hypertension, diabetes, renal failure, and utilization of bioprothesis [5]. Among these, calculating BSA from a patient's height and weight has been suggested as the first in a simple three-step algorithm to determine the type and size of prosthesis according to EOAi's reference values [6]. The aforementioned risk factors can easily be recognized clinically and preoperatively; however, their qualitative nature prevents surgeons from knowing whether further operative considerations, such as aortic root enlargement (ARE), are warranted.

With the above limitations in mind, multimodality imaging has been proposed as a powerful and comprehensive approach to identify and quantitate PPM [7]. 2D/3D transthoracic (TTE) and multidetector computed tomography (CT) remain the most widely used tools to measure the aortic annulus dimensions pre-procedurally [3]. Previous studies have documented the superiority of CT measurements as compared to echocardiographic values and recommended the former to be routinely included in prosthesis sizing [8]. In practice, however, some inconsistencies still exist between calculated values and actual manufacturers' prothesis size. Hence, a more direct and robust imaging-based algorithm would significantly improve the ability to predict PPM before implantation.

Herein, we examined AVR cases from our institution and assessed the correlation between the aortic valve diameter as measured by echocardiography and CT scan preoperatively versus the true size of implanted valve. Our aim is to develop an imaging-based algorithm to predict the prosthesis valve size prior to AVR.

## Methods

### Study design

This retrospective cohort study included patients who underwent AVR at the Department of Cardiovascular Surgery, University Medical Center at Ho Chi Minh City from January 2017 to December 2020. All patients had 128-slice CT scan and TTE and both were used to calculate the aortic annulus.

The aortic annulus is a crown-shaped structure that serves as the insertion point for the aortic cusps. Its highest and lowest points are located at each of the three commissures and between any two of them, respectively. The annulus, which lacks a planar structure, is compressed to the round-shaped prosthesis after conventional AVR. We therefore assumed that prothesis size is correlated with the plane passing all three nadirs of the aortic valve.

**Fig. 1:**
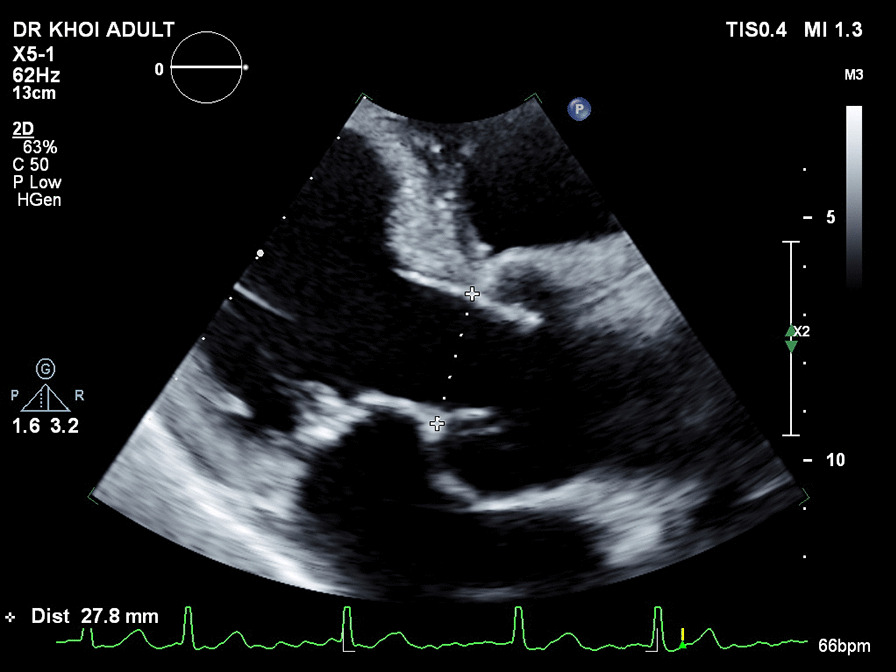
Measurement of aortic annulus diameters on TTE

Using TTE, the diameters of aortic annulus and left ventricular outflow tract (LVOT), as well as the sinus of Valsalva, sinotubular junction (STJ) and ascending aorta, were measured on the parasternal long axis view (PLAX) (Fig. 1). While reproducible, the results were greatly dependent on echocardiographers.

**Fig. 2:**
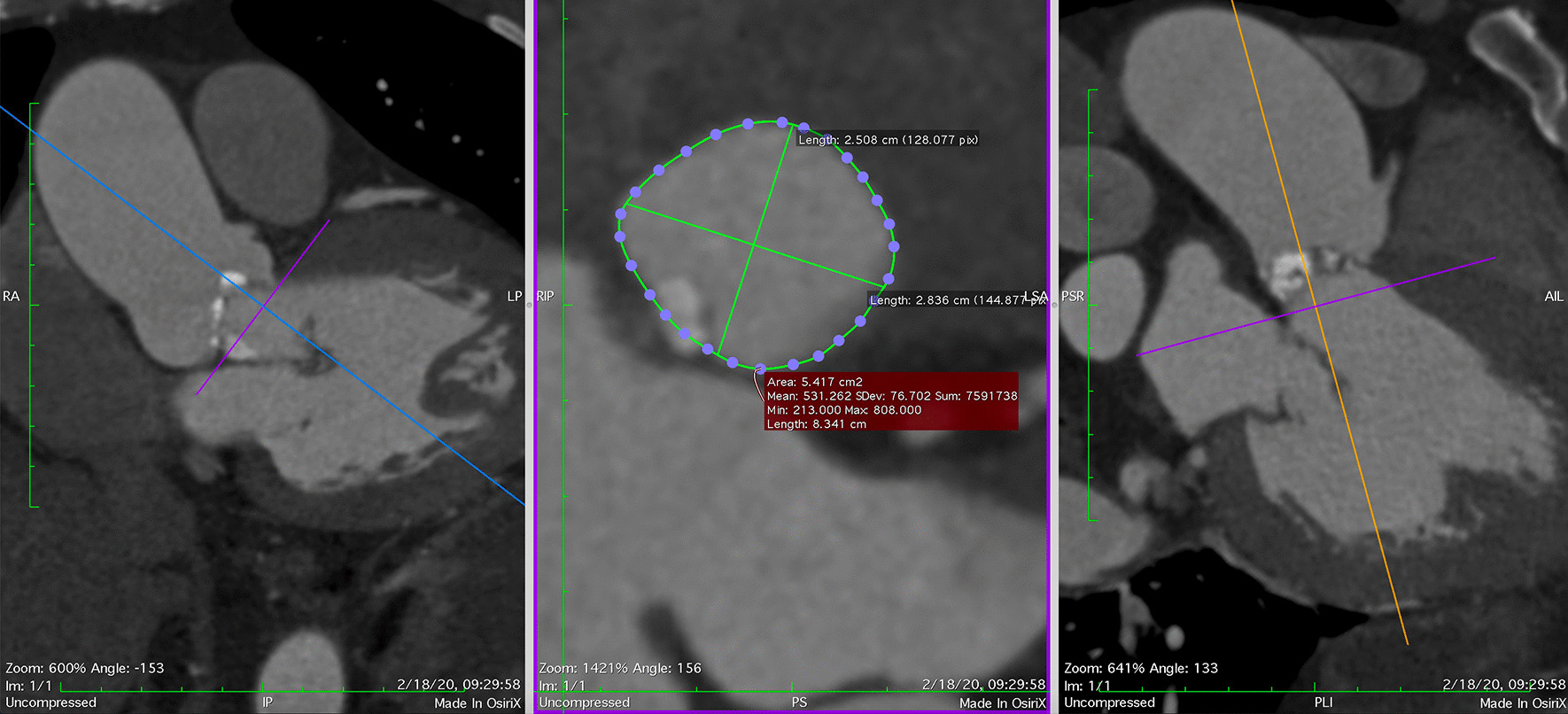
Measurement of aortic annulus diameters on CT scan

On CT scan, we employed multiplanar reconstruction (OsiriX™ software, Bernex, Switzerland) to map out the plane that passes through three nadirs of the aortic valve. The largest, smallest, average, perimeter-derived and area-derived diameters were then calculated (Fig. 2).

All diameters were measured at end-systole.

### Surgical techniques

We performed AVR via three approaches: conventional full sternotomy, upper ministernotomy or second intercostal minithoracotomy. For the minimally invasive procedures, peripheral cardiopulmonary bypass (CPB) was established with femoral vessels. Custodiol® HTK Solution was delivered antegradely into the aortic root or the coronary ostia in patients with severe aortic regurgitation and was repeated every 120 min if necessary. We used single annular sutures for intra-annular valve replacement and ventricular-side pledgeted sutures for supra-annular valve replacement. Pledgeted sutures (supra-annular fashion) were utilized when severe calcifications were found on the valve leaflets and annulus. Finally, transesophageal echocardiography (TEE) was used to assess the surgery results.

For mechanical valve, we used SJM™ Masters Series Mechanical Heart Valve (Abbott Laboratories, Chicago, Illinois, USA). For tissue valve, we used Carpentier-Edwards PERIMOUNT Magna Ease (Edwards Lifesciences, Irvine, California, USA).

### Statistical analysis

Data analysis was performed using MedCalc version 19 (Medcalc Software Ltd, Ostend, Belgium). The associations between imaging measurements and valve size were evaluated using scatter plots and Pearson’s correlation coefficients. Linear regression model was used to predict the surgical sizing from imaging-based values. As strong correlations between parameters were found, we performed univariable linear regression and selected the highest correlated measurements to build the prediction model (outcome = a + b × measurement; a: intercept; b: slope). The 95% confidence interval (CI) of the slope and intercept are reported. We analyzed the whole dataset and then performed subgroup analyses for tissue valve and mechanical valve. Statistically significant p value was set to equal or less then 0.05.

## Results

A total of 144 patients were included in our study. Baseline and operative characteristics are presented in Tables 1 and 2, respectively. Overall, we found no significant differences in the durations of cardiopulmonary bypass, cross-clamp, mechanical ventilation and ICU stay among three surgical techniques. Interestingly, patients undergoing second intercostal minithoracotomy had longer CPB and cross-clamp time but shorter mechanical ventilation length and ICU stay. Regarding adverse outcome, one death occured at postoperative day five due to cerebral hemorrhage. No aortic dissection was recorded. Nine patients required reoperation due to bleeding, including five cases of full sternotomy, three upper ministernotomies and one second intercostal minithoracotomy. Operative approach was changed to full sternotomy in two patients due to uncontrolled bleeding (upper ministernotomy, one case) and poor exposure (second intercostal minithoracotomy, one case).

**Table 1:** Baseline characteristics

Variable	Cohort (n = 144)
*Demographic variables*	
Age (years)	56.2 ± 14.6
Sex (female)	60 (41.6)
Comorbidities, n (%)	
Hypertension	65 (45.1)
Type II diabetes mellitus	31 (21.5)
Preoperative atrial fibrillation	49 (34.0)
*AVR variables*	
Etiology of aortic valve lesions, n (%)	
Post rheumatic	31 (21.5)
Degenerative	51 (35.4)
Congenital	62 (43.1)
Approach	
Full sternotomy	68 (47.2)
Upper ministernotomy	47 (32.6)
Second intercostal minithoracotomy	29 (20.1)
Prosthesis type	
Mechanical valve	62 (43.1)
Tissue valve	82 (56.9)
Valve size (mm), mean ± SD	21.36 ± 1.8
Mechanical valve	21.2 ± 1.77
Tissue valve	21.5 ± 1.74

*AVR* aortic valve replacement

**Table 2:** Intraoperative details

Variable		P value
*Cardiopulmonary bypass time (minutes)*		
Total	116.5 ± 35.1	
Full sternotomy	104.5 ± 36.0	0.281
Upper ministernotomy	108.5 ± 21.7	
Second intercostal minithoracotomy	126.6 ± 25.7	
*Cross-clamp time (minutes)*		
Total	82.7 ± 21.6	
Full sternotomy	69.6 ± 20.2	0.090
Upper ministernotomy	66.5 ± 16.1	
Second intercostal minithoracotomy	92.3 ± 17.5	
*Mechanical ventilation time (hours)*		
Total	36.2 ± 28.6	
Full sternotomy	43.9 ± 30.7	0.112
Upper ministernotomy	31.7 ± 30	
Second intercostal minithoracotomy	23.5 ± 8.9	
*Length of ICU stay (days)*		
Total	3.3 ± 1.4	
Full sternotomy	3.7 ± 1.3	0.49
Upper ministernotomy	2.9 ± 1.7	
Second intercostal minithoracotomy	2.9 ± 1.3	
*Postoperative complications, n (%)*		
Mortality	1 (0.69)	
Permanent stroke	2 (1.4)	
Aortic dissection	0 (0)	
Prolonged mechanical ventilation*	21 (14.6)	
Renal failure requiring dialysis	6 (4.2)	
Reoperation for bleeding	9 (6.3)	
Full sternotomy	5	
Upper ministernotomy	3	
Second intercostal minithoracotomy	1	
Conversion to full sternotomy	2	
Upper ministernotomy (n = 47)	1 (2.2)	
Second intercostal minithoracotomy (n = 29)	1 (6.9)	

*ICU* intensive care unit

*P < 0.05 is considered significantly different

Table 3 illustrates prosthesis size as according to two suture techniques, namely intra-annular single suture and supra-annular pledgeted suture, and three surgical approaches. Neither parameters significantly affect the prosthesis size.

**Table 3:** Prosthesis size according to different suture techniques and surgical approaches

Variables	Prosthesis size	P-value
*Suture technique*		
Intra-annular single suture (n = 73)	21.1 ± 1.7	0.124 (t-test)
Supra-annular pledgeted suture (n = 41)	20.8 ± 1.8	
*Surgical approach*		
Full sternotomy	21.4 ± 1.67	0.349 (One-way ANOVA)
Upper ministernotomy	21.4 ± 1.96	
Second intercostal minithoracotomy	20.7 ± 1.89	

**Fig. 3:**
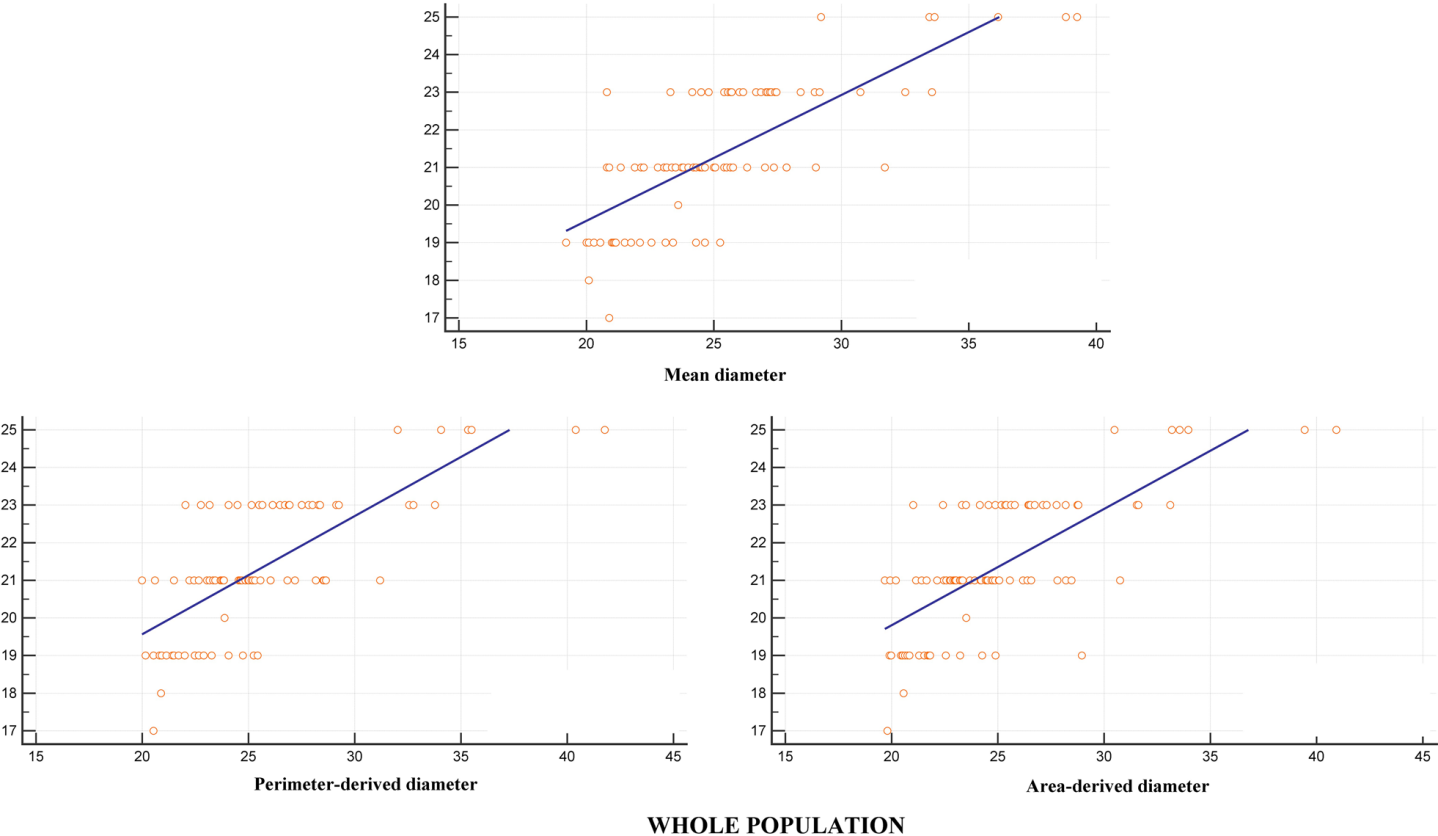
Scatterplots for the correlation of each calculated diameters with prosthesis size

The relationship between diameters calculated by imaging modalities and the implanted prothesis size was computed using Pearson correlation coefficients (Table 4) and graphed as scatterplots (Fig. 3). TTE-measured diameters showed the weakest correlation, whereas those calculated from CT scan were strongly correlated with surgical prosthesis sizing (coefficients were greater than 0.70 for all diameters and for both mechanical and tissue valve). Hence, CT-based predictive model of aortic prothesis size were developed using univariable linear regression (Table 5).

**Table 4:** Pearson correlation coefficients between imaging-measured diameters and intraoperative prothesis size

Imaging modality	Calculated aortic annulus diameters		
	All patients	Mechanical valve	Tissue valve
TTE diameter (PLAX diameter)	0.59	0.65	0.49
CT scan			
Mean diameter	0.79	0.79	0.79
Perimeter-derived diameter	0.76	0.77	0.76
Area-derived diameter	0.75	0.73	0.76

*CT* computed tomography, *PLAX* parasternal long axis view, *TTE* transthoracic echocardiography

**Table 5:** CT scan-based prediction model of aortic prosthesis size

Calculated diameter	All patients	Mechanical valve group	Tissue valve group
Mean diameter	12.89 + 0.335 × d (R^2^ = 0.6241)	11.104 + 0.378 × d (R^2^ = 0.6241)	13.068 + 0.341 × d (R^2^ = 0.6241)
Perimeter-derived diameter	13.275 + 0.315 × d (R^2^ = 0.5776)	11.213 + 0.371 × d (R^2^ = 0.5929)	13.724 + 0.308 × d (R^2^ = 0.5776)
Area-derived diameter	13.626 + 0.309 × d (R^2^ = 0.5625)	11.593 + 0.363 × d (R^2^ = 0.5329)	13.910 + 0.310 × d (R^2^ = 0.5776)

*CT* computed tomography, *d* corresponding calculated diameter, *R*^2^ R-squared, coefficient of determination of the model

## Discussion

The major finding of this study is that CT-calculated aortic annulus diameter is more reliable than TTE preoperatively and hence could be used to develop a predictive model of prosthesis size, eventually preventing PPM.

Several preventive strategies can be considered when PPM following AVR is likely [3]. A newly developed generation of prosthesis, the stentless bioprosthesis, is a valve designed for supra-annular implantation [9]. The new era of transcatheter AVR (TAVR) also promised to lower the prevalence of PPM, with supporting data from Asian population [10, 11]. Nevertheless, these valves might not be commercially available or too costly for patients in developing countries. Under these circumstances, aortic root enlargement (ARE) is commonly indicated to place a larger valve and has been performed for many years. However, not only does this practice add to operation time and complexity, but could also negatively affect morbidity and mortality in the field of minimally invasive surgical AVR, such as mini-thoracotomy and upper ministernotomy [12]. In addition, ARE is associated with several complications, such as mitral valve prolapse and aorto-left atrial fistula [13]. Therefore, recognizing the need for ARE and adequately determining a patient's risk of PPM are crucial prior to AVR.

This study compared preoperative CT scan and TTE measurements of aortic annulus diameter to intraoperative annular sizing. Our results agree with previous literature, which indicated that CT-based calculations were better correlated with operative values. In particular, Kempert et al. demonstrated that utilizing the “effective” diameter on CT scan is preferable to TTE values in patients with oval-shaped annulus [14]. Likewise, Daskevish et al. found that the calculated annular measurements with CT are closer to operative sizing with a Hegar dilator [8]. While suggesting that imaging modalities (CT and TTE) could be fairly accurate in predicting the aortic annulus size, both studies shared similar limitations, i.e. small sample size (26–33 patients) and did not establish predictive models. A recent review by Pibarot et al. proposed an algorithm to predict and prevent PPM by using multidetector CT or 3D TEE but still did not provide an estimated prothesis size preoperatively [3]. This could lead to unplanned ARE during AVR and pose major challenges to inexperienced surgeons.

The superiority of CT scan over echocardiography can be explained as follows. TTE is echocardiographer-dependent, and a single dimension on PLAX cannot represent the whole aortic annulus. On the other hand, CT scan provides three different diameters (mean, perimeter-derived and area-derived diameter) that are fairly comparable when calculating the prosthesis size preoperatively. In practice, however, echocardiography is non-invasive and presents as the only option when CT scan is contraindicated or unavailable. Hence, measurements predicted from TTE alone could still be considered while bearing in mind that they might not be as accurate as CT scan-based. Our study showed that CT-calculated algorithms for mechanical and tissue valve shared similar coefficients, thus proving CT-based predictor as applicable for both prothesis types. Overall, we recommend combining all three diameters on CT scan to minimize possible errors during measurements.

To our knowledge, this is the first study to propose a quantitative, imaging-based model to predict PPM prior to AVR. Knowing each patient's predictive prosthesis size, surgeons can anticipate and develop suitable strategies (eg using valves with larger EOAi, preparing for ARE or performing TAVR in lieu of surgical AVR) before incision. This would in turn help decrease morbidity caused by unintended procedures and lead to better patient outcome.

The main limitations of this study are its retrospective nature, small sample size and that few types of prostheses were used to establish the predictors. As different manufacturers produce protheses with similar size but largely inconsistent dimensions, the two most popular prostheses in our center were chosen to increase the model accuracy (SJM™ Masters Series for mechanical valve and Carpentier-Edwards PERIMOUNT Magna Ease for bioprosthesis). As a result, the suggested algorithm might only apply to these two and further studies using a wider variety of prothesis types are needed.

## Conclusions

Preoperative CT scan measurements is efficient in predicting the size of aortic prosthesis. If CT is contraindicated or unavailable, TTE is an alternative imaging method, though its dependence on echocardiographers and inability to represent the full aortic annulus limit its accuracy. Together, these two imaging modalities can be incorporated into a quantitative and straightforward algorithm to predict PPM preoperatively.

## Data Availability

The datasets used and/or analysed during the current study are available from the corresponding author on reasonable request.

## Abbreviations

AVR: Aortic valve replacement
ARE: Aortic root enlargement
PPM: Patient-prosthesis mismatch
CPB: Cadiopulmonary bypass
TAVR: Transcatheter aortic valve replacement
ICU: Intensive care unit
CT: Computed tomography
TTE: Transthoracic echocardiography
TEE: Transeophageal echocardiography
PLAX: Parasternal long axis
LVOT: Left ventricular outflow tract
EOA: Effective orifice area
